# Implementation of test-and-treat with doxycycline and temephos ground larviciding as alternative strategies for accelerating onchocerciasis elimination in an area of loiasis co-endemicity: the COUNTDOWN consortium multi-disciplinary study protocol

**DOI:** 10.1186/s13071-019-3826-8

**Published:** 2019-12-04

**Authors:** Samuel Wanji, Theobald Mue Nji, Louise Hamill, Laura Dean, Kim Ozano, Abdel J. Njouendou, Raphael A. Abong, Elisabeth Dibando Obie, Andrew Amuam, Relindis Ekanya, Winston Patrick Chounna Ndongmo, Bertrand L. Ndzeshang, Ebua Gallus Fung, Dum-Buo Nnamdi, Desmond Akumtoh Nkimbeng, Samuel Teghen, Emmanuel Kah, Helen Piotrowski, Armelle Forrer, Jahangir A. M. Khan, Maame E. Woode, Louis Niessen, Victoria Watson, Zakariaou Njoumemi, Michele E. Murdoch, Rachael Thomson, Sally Theobald, Peter Enyong, Joseph D. Turner, Mark J. Taylor

**Affiliations:** 1 COUNTDOWN, Department of Microbiology and Parasitology, Faculty of Science, University of Buea, P.O. Box 63, Buea, Cameroon; 2 COUNTDOWN, Research Foundation for Tropical Diseases and Environment, P.O. Box 474, Buea, Cameroon; 3 COUNTDOWN, Department of Sociology and Anthropology, Faculty of Social and Management Sciences, University of Buea, P.O. Box 63, Buea, Cameroon; 4 COUNTDOWN, Department of International Public Health, Liverpool School of Tropical Medicine, Pembroke Place, Liverpool, UK; 5 COUNTDOWN, Department of Biomedical Sciences, Faculty of Health Sciences, University of Buea, P.O. Box 12, Buea, Cameroon; 6 COUNTDOWN, Department of Tropical Disease Biology, Liverpool School of Tropical Medicine, Pembroke Place, Liverpool, UK; 7 COUNTDOWN, Department of Clinical Sciences, Liverpool School of Tropical Medicine, Pembroke Place, Liverpool, UK; 8 Sightsavers, 35 Perrymount Road, Haywards Heath, UK; 9 Centre for Health Economics, Monash University, Victoria, Australia; 10 Department of International Health, Johns Hopkins School of Public Health, Baltimore, USA; 11 Health Economics Unit, Department of Public Health, Faculty of Medicine and Biomedical Sciences, University of Yaounde I, P.O. Box 1364, Yaounde, Cameroon; 12 West Herts Hospitals NHS Trust, Watford General Hospital, Vicarage Road, Watford, UK

**Keywords:** Onchocerciasis, Onchodermatitis, Wolbachia, Doxycycline, Cameroon, Vector Control, Temephos, Abate, NTD Elimination, Multi-disciplinary

## Abstract

**Background:**

Onchocerciasis is a priority neglected tropical disease targeted for elimination by 2025. The standard strategy to combat onchocerciasis is annual Community-Directed Treatment with ivermectin (CDTi). Yet, high prevalence rates and transmission persist following > 12 rounds in South-West Cameroon. Challenges include programme coverage, adherence to, and acceptability of ivermectin in an area of *Loa loa* co-endemicity. Loiasis patients harbouring heavy infections are at risk of potentially fatal serious adverse events following CDTi. Alternative strategies are therefore needed to achieve onchocerciasis elimination where CDTi effectiveness is suboptimal.

**Methods/design:**

We designed an implementation study to evaluate integrating World Health Organisation-endorsed alternative strategies for the elimination of onchocerciasis, namely test-and-treat with the macrofilaricide, doxycycline (TTd), and ground larviciding for suppression of blackfly vectors with the organophosphate temephos. A community-based controlled before-after intervention study will be conducted among > 2000 participants in 20 intervention (Meme River Basin) and 10 control (Indian River Basin) communities. The primary outcome measure is *O. volvulus* prevalence at follow-up 18-months post-treatment. The study involves four inter-disciplinary components: parasitology, entomology, applied social sciences and health economics. Onchocerciasis skin infection will be diagnosed by skin biopsy and *Loa loa* infection will be diagnosed by parasitological examination of finger-prick blood samples. A simultaneous clinical skin disease assessment will be made. Eligible skin-snip-positive individuals will be offered directly-observed treatment for 5 weeks with 100 mg/day doxycycline. Transmission assessments of onchocerciasis in the communities will be collected post-human landing catch of the local biting blackfly vector prior to ground larviciding with temephos every week (0.3 l/m^3^) until biting rate falls below 5/person/day. Qualitative research, including in-depth interviews and focus-group discussions will be used to assess acceptability and feasibility of the implemented alternative strategies among intervention recipients and providers. Health economics will assess the cost-effectiveness of the implemented interventions.

**Conclusions:**

Using a multidisciplinary approach, we aim to assess the effectiveness of TTd, alone or in combination with ground larviciding, following a single intervention round and scrutinise the acceptability and feasibility of implementing at scale in similar hotspots of onchocerciasis infection, to accelerate onchocerciasis elimination.

## Background

Onchocerciasis (river blindness) is a vector-borne, neglected tropical disease (NTD), caused by the filarial parasite *Onchocerca volvulus*. Physical manifestations include troublesome itching, skin rash, visual impairment and irreversible blindness [1–3]. Currently, the World Health Organization (WHO) through the African Programme for Onchocerciasis Control (APOC) and, latterly, The Extended Special Programme for the Elimination of NTDs (ESPEN), is using mass drug administration (MDA) with ivermectin (IVM) to move towards disease elimination. Ivermectin is a microfilaricidal drug that kills the immature larval form of the parasite, microfilariae (mf) found in the skin, to prevent disease and transmission to the blackfly vector [4, 5]. Based on predictive model simulations, the WHO recommends that annual MDA for at least 15–17 years with high population coverage (80%) may impact on transmission and thus abate incidence of skin infection in younger individuals [1, 6]. MDA has been conducted in the South-West region of Cameroon for more than 12 years in a strategy known as Community-Directed Treatment with IVM (CDTi), yet higher than expected prevalence and intensity of onchocerciasis persists including in children born in the CDTi period [7–9].

The persistence of onchocerciasis in South-West Cameroon highlights that a transition from control to elimination of this disease using CDTi is likely to be complicated by multiple factors. Of major concern is the geographical overlap with the related filarial, *Loa loa*, which is a risk factor for incidence of severe (neurological) adverse events (SAE), as well as more frequent non-neurological adverse events (AE), post-CDTi [10, 11]. The perception of IVM-related SAE may impinge on adherence to CDTi even in areas free from *L. loa* [9]. Therefore, an urgent need exists to validate and implement alternative strategies to accelerate elimination of onchocerciasis, as well as to more fully understand structural and health system factors and social and contextual reasons for sub-optimal success of the CDTi strategy so that these may be avoided in the future, especially in areas of *L. loa* co-endemicity.

The objective of this research study is to assess the effectiveness, acceptability and feasibility of a test-and-treat strategy for onchocerciasis control using the WHO endorsed macrofilaricidal treatment, doxycycline (Test & Treat with doxycycline; TTd), a potential alternative for IVM. Doxycycline (DOX) differs from IVM as it targets the filarial symbiont, *Wolbachia*, which sterilises adult worms residing in human tissues, preventing the production of mf seeding the skin and also significantly reduces adult lifespan to mediate macrofilaricidal activity within two years post-treatment [12, 13]. Further benefit of depleting *Wolbachia* in existing skin mf is the inhibition of development to infectious stage larvae within the blackfly vector, thus more immediately impacting on transmission [14]. Doxycycline does not cause rapid microfilaricidal activity, and its target, *Wolbachia*, is not present in *L. loa* [15–17]. Therefore, DOX does not trigger IVM-like inflammatory AE and is completely safe in the treatment of onchocerciasis/loiasis co-infection. Our hypothesis is that by offering an alternative treatment to IVM, community members who are reluctant to engage with the CDTi approach due to factors listed above will be able to access treatment for onchocerciasis. In addition, the recent success of ground larviciding to reduce biting incidence of the *O. volvulus* vector, *Simulium* (blackfly), will be implemented in addition to TTd to evaluate if there is added benefit in combining alternative strategies [18–20].

## Methods/design

### Study design and objectives

The implemented study is a community-based controlled longitudinal intervention study which consists of a baseline survey and a follow-up survey 18 months after the commencement of the interventions. The interventions consist of three study arms: CDTi approach as normal (Study Arm 1); testing and treating community members with DOX alone (Study Arm 2); or in combination with temephos (abate) ground larviciding (Study Arm 3). Additionally, all study participants will be advised to adhere to CDTi unless their diagnosis results for *L. loa* indicate a risk of loiasis SAE. Control community clusters (Study Arm 1) will continue the standard control strategy only (CDTi). A quantitative approach will be used to assess the effectiveness of the implemented interventions and qualitative research methods including interviews, focus-group discussions, observations of the test-and-treat process and implementer field diaries reflecting on the research will be used to assess the perceptions and attitudes towards CDTi, as well as the acceptability and feasibility of the implemented interventions amongst community drug distributors (CDDs) and study participants of TTd and ground larviciding communities.

The objectives of this study are to:

(i) Determine the major community participant and community drug distributor attitudes associated with sub-optimal effectiveness of the current CDTi strategy;
(ii) Assess the acceptability and feasibility of implementing alternative strategies for onchocerciasis elimination (namely TTd and ground larviciding);
(iii) Determine the effectiveness of implementing TTd and ground larviciding in accelerating focal elimination of onchocerciasis.

### Study area and site selection

The study is conducted in the Rumpi Hills region, South-West Cameroon. The area is characterized by a volcanic ridge culminating at 1764 m from which the Meme, Mungo, Manyu, and Ndian rivers take their waters. These fast-flowing rivers provide perennial breeding sites for the major *O. volvulus* blackfly vector, *Simulium damnosum* (*s. l.*), leading to continuous onchocerciasis transmission.

The climate is characterized by high temperatures (25–32 °C), heavy rainfall (2500–4000 mm) for seven months of the year (April-November) and a short dry season from December to March. An evergreen and humid forest is the main feature of this zone although it is gradually being replaced by plantations (rubber, palm oil, cocoa, coffee, food crops) [21].

Onchocerciasis prevalence data collected in 2012 indicated ongoing transmission in the Meme, Mungo and Manyu river basins [7]. The Meme basin exhibited the highest mean skin mf prevalence (52.7%) with prevalence rates above 40% in 13/16 communities examined, as well as the highest intensity of infection and prevalence of nodules [7]. This area was selected so implementing alternative strategies could have most impact.

**Fig. 1:**
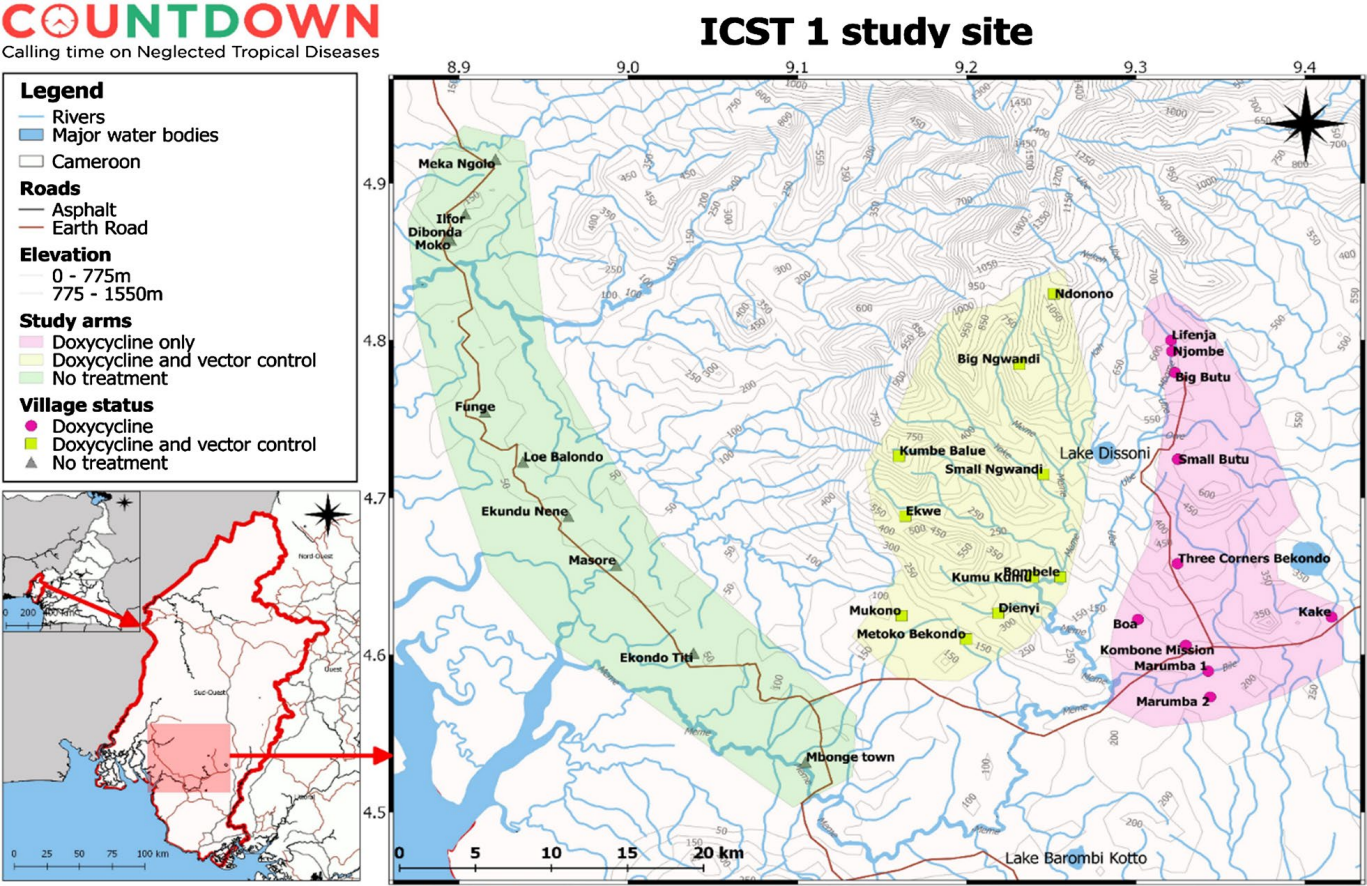
Map of the study area indicating spatial segregations of intervention community clusters

Each study intervention arm (Arm 2: DOX only and Arm 3: DOX+VC) will include 10 communities located in the Meme River Basin. The 10 communities of the control arm (Arm 1) will be selected from the nearby Ndian River Basin. Study sites are displayed in Fig. 1. Community selection was based on hydrological mapping, to achieve maximum spatial segregation between vector control and non-vector control study sites.

### Pre-study processes

#### Advocacy and stakeholder engagement

Advocacy and stakeholder engagement meetings will be organised and held at different levels of the health system from the national to the community level. They will involve key health system stakeholders and community leaders who will communicate the information to their respective areas of influence and communities. A key issue will be to collect baseline parasitological data before CDTi takes place in the study communities.

#### Community entry and sensitisation

As a first entry point to the communities, community leaders will be invited to all stakeholder and advocacy meetings. Following this, community leaders and health systems stakeholders will be engaged in sensitising other community members in all 20 intervention communities.

#### Training of health workers and CDDs

The district and health area staff will be trained by the research team, and the Chiefs of Health Centres will cascade the training to CDDs, community leaders, religious leaders, and Community Based Organisations. Training activities will be supervised and monitored by the health district staff and the research team. CDDs will receive TTd specific training, which will include drug packaging, directly observing treatment with DOX, filling of registers, following up of participants, monitoring side effects, managing minor side effects and evaluating the entire process.

### Study participants

#### Population census

A community census will be carried out in all 30 study communities to evaluate the population eligible for screening. All individuals aged 5 years and above and who will have lived in the community for five years or more at the time of the census will be eligible for the parasitological screening.

#### Enrolment in the intervention study and eligibility criteria

All censused individuals will be explained the objectives and procedures of the intervention study and informed assent/consent will be obtained before enrolment. For children and adolescents, assent will be obtained using information sheets specifically designed for children (age below 15 years) and adolescents (age 15–18 years) and consent will be obtained from a parent or legal guardian.

Participants enrolled in all study arms will be tested for *O. volvulus* and *L. loa* infection. Only microfilaridermic (individuals diagnosed with *O. volvulus* infection), who consent to participate and meet the eligibility criteria to take DOX will be enrolled in the intervention study. Children below 9 years-old, pregnant and breast-feeding women, individuals suffering from any chronic disease (hypertension, diabetes, tuberculosis, HIV/AIDS) and those on other daily medications will be excluded from DOX treatment due to potential adverse reactions. All microfilaridermic women of childbearing age (15–49 years) will be tested for pregnancy, with their consent, using hCG Urine Pregnancy Test Strip (LabACON, Lancing, UK). Women with a negative test will be advised to avoid any occurrence of pregnancy during treatment (abstinence or contraceptive use). Results of pregnancy testing will be communicated verbally to female patients in private.

#### Participants in the qualitative assessments

Community members, health system implementers of TTd and ground larviciding implementers will be recruited for semi-structured interviews and focus-group discussions. Table 1 summarises the planned number of qualitative interviews.

**Table 1:** Summary of qualitative interviews

Group interviewed	Study Arm 2		Study Arm 3		Total
	Men	Women	Men	Women	
Refusers at screening phase	2–4	2–4	2–4	2–4	8–16
Onchocerciasis negative and refuse ivermectin	2–4	2–4	2–4	2–4	8–16
Refusers of doxycycline	2–4	2–4	2–4	2–4	8–16
Acceptors of doxycycline	6–12	6–12	6–12	6–12	24–48
Post-training interviews with CDDs	2–4	2–4	2–4	2–4	8–16
Post-intervention interviews with CDDs	2–4	2–4	2–4	2–4	8–16
Post-training interviews with chief of centre	2–3		2–3		4–6
Post-intervention interviews with chief of centre	2–3		2–3		4–6
Key Informant interviews with influential community members	4–8		4–8		8–16
Ground larviciding implementers	na		4–8		4–8

*Abbreviation*s: CDD, community drug distributor; na, not applicable

Interviews will take place to assess the perception and acceptability of the CDTI strategy and the new TTd strategy. Interview participants will be representative of community members participating in the census who refused to be screened (skin-snipped), *O. volvulus-*negative participants who refuse CDTi, *O. volvulus-*positive participants who either refused or accepted DOX, as well as influential community members (key informants). CDDs and Chiefs of Health Centres will be interviewed to investigate perceptions of the TTd strategy from a health system implementers perspective.

#### Participants in the health economic analysis

A purposive sampling technique will be used to enrol households eligible for outcome assessments. A sampling frame of all eligible participants will be obtained from CDD and community health workers.

### Treatment procedure

All enrolled participants will be treated with 100 mg DOX daily for five weeks. DOX capsules will be procured from a UK wholesale pharmaceutical supplier and shipped from UK at ambient temperature, to be used within 6 months.

The treatment will be delivered using an enhanced CDTi approach under direct supervision [22]. A maximum of 10 patients will be allocated to each CDD for daily administration. CDDs will be compensated for earnings missed during the treatment delivery period by payment of 1000 CFA per day. Treatment will be taken with a food provided by the research team, to avoid potential gastric side effects to DOX. Participants shall swallow the DOX capsule in the presence of the CDD (directly observed treatment) and treatment uptake will be recorded in registers. If enrolled individuals are absent due to work or other commitments, capsules will be supplied to them, and empty blister packs returned to the community health implementer for monitoring of adherence. A member of the research team will be assigned to each community to supervise treatment activities of the CDDs.

**Fig. 2:**
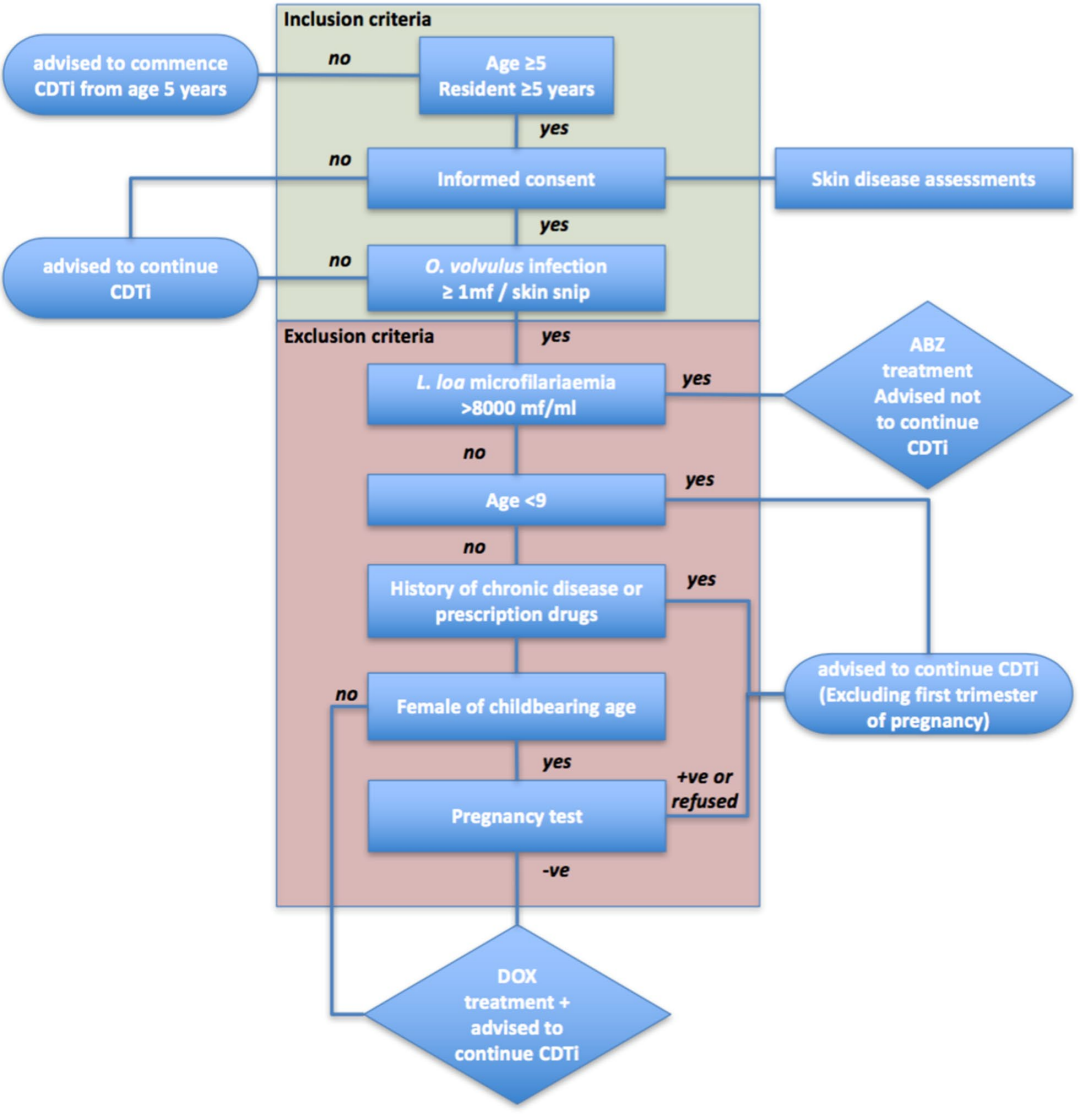
Doxycycline treatment decision tree with inclusion and exclusion criteria. *Abbreviations*: ABZ, albendazole; CDTi, community directed treatment with ivermectin; DOX, doxycycline; mf, microfilariae; ml, millilitre

All diagnosed onchocerciasis patients and/or loiasis patients will be verbally informed of their infection status. Irrespective of eligibility to DOX treatment, patients will be advised to adhere to CDTi unless they are diagnosed with *L. loa* microfilaremia > 8000 mf/ml. Any such patient will be offered a 400 mg albendazole treatment. The flow chart (decision tree) of the test-and-treat with DOX is as shown in Fig. 2.

#### Assessment of doxycycline-related adverse events

Clinical monitoring of adverse reactions (AEs) will be undertaken by community health workers throughout the 5-week period of DOX treatment under the supervision of the research monitors and the chief of the local health centres. Patients will be asked by questionnaire for any side effects of the drugs as per protocol. AEs will be graded as absent, mild, moderate or severe. Individuals will be asked to report any signs and symptoms that were not experienced prior to drug administration. All symptoms will be documented in patients’ treatment cards and medication or hospitalisation will be provided where necessary.

### Quantitative assessments

#### Demographic and treatment intake history assessments

All censused participants will be asked to answer a structured questionnaire to collect data on socio-demographics, self-reported adherence to CDTi and albendazole intake history.

For the control communities, a minimum census survey recording age and gender will be conducted to keep interactions with, and interference by the study at a minimum.

#### Parasitological assessments

- *Onchocerciasis testing.* Onchocerciasis diagnosis will be offered *via* the gold-standard method of skin biopsy (skin snipping). Two bloodless skin biopsies will be taken from the left and right iliac crest of each participant using a sterile 2 mm corneo-scleral punch (CT 016 Everhards 2218-15 C, Meckenheim, Germany). Each participant skin sample will be incubated in 2 separate wells of a sealed microtiter plate containing 2 drops of normal saline for 24 h. The medium will be examined under a light microscope. Emerged mf will be counted and expressed per biopsy (which corresponds approximately to 1 mg of skin; mf/mg) [7, 23]. The skin biopsies and any emergent mf will be placed into 1.5 ml Eppendorf tubes and 80% ethanol added and stored at −80 °C for subsequent use (DNA extraction).
- *Loiasis and other blood-borne filariae testing.* Study participants will undergo a finger prick blood test for *L. loa*, to quantify the potential for loiasis adverse reactions to the standard CDTi treatment only, as *L. loa* is not impacted by DOX [13]. A 50 µl blood smear will be prepared, dehaemoglobinised for 5–15 min, and fixed with methanol. Smears will be stained in 10% Giemsa for 45 min and allowed to air-dry [24]. Microfilariae of *L. loa* and/or *M. perstans* will be identified using microfilarial identification keys [25]. Slides will be read by trained technicians and counts will be expressed as the number of microfilariae per millilitre (mf/ml) of blood.

#### Onchodermatitis clinical assessments

Enrolled participants will undergo a clinical skin assessment conducted by nursing staff from local district hospitals, using a standardised clinical classification and grading system for onchodermatitis [26]. The nurses will be specifically trained by an expert dermatologist in onchocercal skin disease assessment and coding. Each assessor will be evaluated by inter-observer variation kappa analysis after conducting examinations on a cohort of 20 individuals with known onchodermatitis manifestations. Assessors with moderate or higher agreement with the trainer (kappa 0.41–0.6 and above) in diagnosing skin disease type will be utilised in enrolment surveys. Clinical assessments will be conducted in a private room within the community, for example within health centres or schools. The aim of skin assessments is to evaluate burden of onchodermatitis in the communities and evaluate effectiveness of CDTi in prevention of onchodermatitis in younger age-groups, related to adherence/participation in CDTi administrations.

#### Monitoring of doxycycline treatment efficacy

As an early surrogate biomarker of long-term treatment efficacy, a sub-set of 50 individuals who have ≥ 10 mf/mg skin pre-treatment and have adhered to 5-week treatment with DOX will be selected. Four months post-commencement of treatment, individuals will be sampled for mf by skin biopsy. Numbers of mf and the number of *Wolbachia* per mf will be determined by quantitative polymerase chain reaction (PCR) [27].

#### Sample size determination

The planned sample size is 800 participants per study arm and 10 community clusters per arm. The sample size was estimated using a cluster sampling approach assuming a baseline mf prevalence of 52.7% based on a survey conducted in the Meme River Basin in 2012, a power of 80%, a precision of 5% and a minimum post treatment reduction in community prevalence of skin microfilaria of 37% [7, 27].

Assuming a population of 2000 individuals and an average of about 65 participants per community, it was estimated that 685 individuals would be assessed against inclusion and exclusion criteria for enrolment into the intervention arms. This number was rounded up to 800, given that 65 is only the average number of people per cluster and that the population size has likely increased between 2012 and 2016. The number of clusters was estimated using the Hayes & Bennet formula assuming 65 individuals per cluster, a power of 80%, a precision of 5% and a between-cluster coefficient estimated to be 0.27, and was further inflated by 20% to allow for loss to follow up and adjustment for potential confounders at baseline, resulting in a number of 10 clusters per arm.

#### Quantitative data management

Data on population census of study communities will be collected using the ODK (Open Data Kit, July 2010, http://opendatakit.org) android application pre-installed in Samsung Galaxy Tablets. A data collection template will be designed in ODK to collect demographic information of community members. After house to house registration of all members within different households (permanent residents), information will be uploaded to a cloud server daily for backup and later export to Microsoft Excel (2013).

During the parasitological/clinical screening phase, eligible study participants will be registered using either a template (for parasitology) designed in the Epi info version 3.5.2 (EpiData Association, Odense, Denmark) android application pre-installed in Samsung Galaxy Tablets or paper record forms (clinical examinations). Baseline parasitology data will be exported to Microsoft Excel (2013). Census and parasitology databases will be combined before exportation to a statistical software.

Participant paper clinical records will be stored in locked cabinets with restricted access at the University of Buea, Cameroon. Electronic participant records will be saved at University of Buea on password-protected laptop PC and backed up daily onto password-protected external hard disk drive which will be stored in a locked cabinet with restricted access and will be encrypted and stored on a highly-secured password protected server at the LSTM. Data will be stored for 10 years after the end of the study.

#### Quantitative data analysis

The primary outcome is the change in *O. volvulus* prevalence (assessed by the presence of skin mf) 18 months after the start of the treatment. Prevalence rates will be reported for each study arm, i.e. TTd only (*n *= 10 communities), TTd + VC (*n *= 10 communities) and the standard care group (*n* = 10 communities), as well as for each study community. The impact of each intervention (TTd only and TTd + VC) on *O. volvulus* infection status at follow-up will be assessed using a multivariate mixed effects logistic regression model with intervention as a fixed effect and community as a random effect. The model will adjust for potential imbalanced participants characteristics between the study arms and potential individual-level or village-level confounders such as age, gender, occupation, or proximity to fast-flowing water sources.

The secondary outcome is intensity of *O. volvulus* infection, defined as skin microfilaridermia, and will be recorded as the number of mf per skin snip at baseline and at two-year follow-up. As microfilaridermia typically follows a negative binomial distribution in an endemic cohort, the impact of treatment on the intensity of skin mf infection will be analysed similarly to infection status but using multivariate mixed effect model accounting for overdispersion such as the Negative Binomial regression.

All statistical analysis will be conducted with input and guidance from members of the LSTM clinical tropical trials unit.

### Entomological assessments

#### Site survey: identification of S. damnosum breeding sites and determination of treatment points

The Meme River and its tributaries (Meme River Basin) will be surveyed to identify breeding sites harbouring *S. damnosum* larvae and to collect adult flies to determine biting rates. Any mature larvae (6 and 7 stages) found will be preserved in Carnoy solution for cytotaxonomic studies. All identified breeding sites will be georeferenced using a global positioning system (GPS). The coordinates will be used to generate a topographical map of the breeding sites.

#### Larval susceptibility testing

The larvicide temephos (Abate® 500 EC-BASF, Douala, Cameroon), will be diluted to give a series of concentrations ranging between 0.5–0.0005 mg/l. Larvae of *S. damnosum* will be collected from several representative breeding sites in the Meme River Basin. The larvae will be exposed to the different concentrations of temephos for a period of 3 h after which mortality will be determined using the WHO criteria [28–30].

#### Blackfly collection

Human landing catches, the current gold standard, will be used to collect blackflies for morphological identification, dissection and calculation of entomological indices (biting, parous, infection and infective rates including transmission potentials) [31]. Collectors will be informed of the risk of exposure to onchocerciasis and will receive prophylactic treatment with doxycycline (100 mg/day) during the collection period and for two weeks following collection (up to a total period of 6 weeks). Collections will be conducted daily for up to five days at two geographically separate collection sites within the TTd or CDTi community areas [32]. Teams comprising two collectors will work at each site for a 5- and 6-hour period, with one collector working from 7:00 h to 12:00 h and the second collector working from 12:00 h to 18:00 h. The location and time will be rotated among collectors to reduce any bias in exposure risk [33].

#### Ground larviciding

The Onchocerciasis Control Programme (OCP) standard larviciding concentration of 0.3 l of Abate/m^3^ of flow rate will be used. Initial assessments of the “larvicide carry”, i.e. the distance downstream where the larvicide remains effective, will determine the number of river/tributary treatment points. It is envisaged that the larviciding will proceed for ≥ 4 weeks depending on impact on *Simulium* larvae but will not exceed 10 weeks. Ground larviciding will be stopped when the biting rate goes below 5 bites/person/day.

#### Monitoring impact on adult and larval blackfly population

Weekly adult blackfly catches will be done using human landing catches as described above. Collections shall begin two weeks before the start of larviciding and continue weekly at the same catching points until the completion of larviciding. Twenty-four hours after each larviciding exercise, all known breeding sites will be checked for the presence or absence of larvae to ascertain the efficacy of the larvicide.

#### Monitoring impact on non-target fauna

There will be both short and long-term monitoring of effect of temephos implementation over 4–10 weeks on the non-target fauna including invertebrates (drifting, benthic) such as shrimps and crabs, and vertebrates including, tadpoles and fish, commonly *Barbus* and *Clarias.* Before larviciding, fishermen/women will be contacted to find out the composition of their regular catches (fish, tadpoles, shrimps, crabs) and these will constitute the reference non-target fauna. This protocol will keep to WHO/OCP standards by selecting 3 dosing points for monitoring at each treatment site. The first point (non-treated zone) will be 500 m upstream from the treatment point, the second and third will be 100 m and 500 m downstream, respectively. Monitoring will be done one-week before larviciding and at 5- and 9-weeks post-larviciding. Two further monitoring rounds will be carried out at 6- and 12-months post-larviciding.

#### Entomological data management

Flies collected at different hours of the day will be recorded singly on designed data collection sheets. Each fly will be dissected, and its physiological age and infection status recorded. Each fly information will be keyed in a template designed in EPI info version 3.5.2 (EpiData Association, Odense, Denmark) and exported to Microsoft Excel (2013) for cleaning and subsequent exportation for analysis with a statistical software.

Entomological indices will be calculated as follows:

- Monthly biting rate: (No. of flies × No. of days in month)/No. of fly collection days
- Monthly transmission potential: (No. of days in month × No. of infectious larvae)/No. of fly collection days × (No. of flies collected /No. of flies dissected)

Monthly biting rate and monthly transmission potential will be compared longitudinally (baseline *vs* 2-year follow-up) using paired t-tests. Further modelling approaches will be developed in consultation with leading experts on blackfly control. Both internal and external vector control specialists have been invited to advise on logistical aspects and data analysis of this part of the study.

All statistical analyses will be conducted with input and guidance from members of the LSTM Clinical Tropical Trials Unit.

### Qualitative assessments

**Fig. 3:**
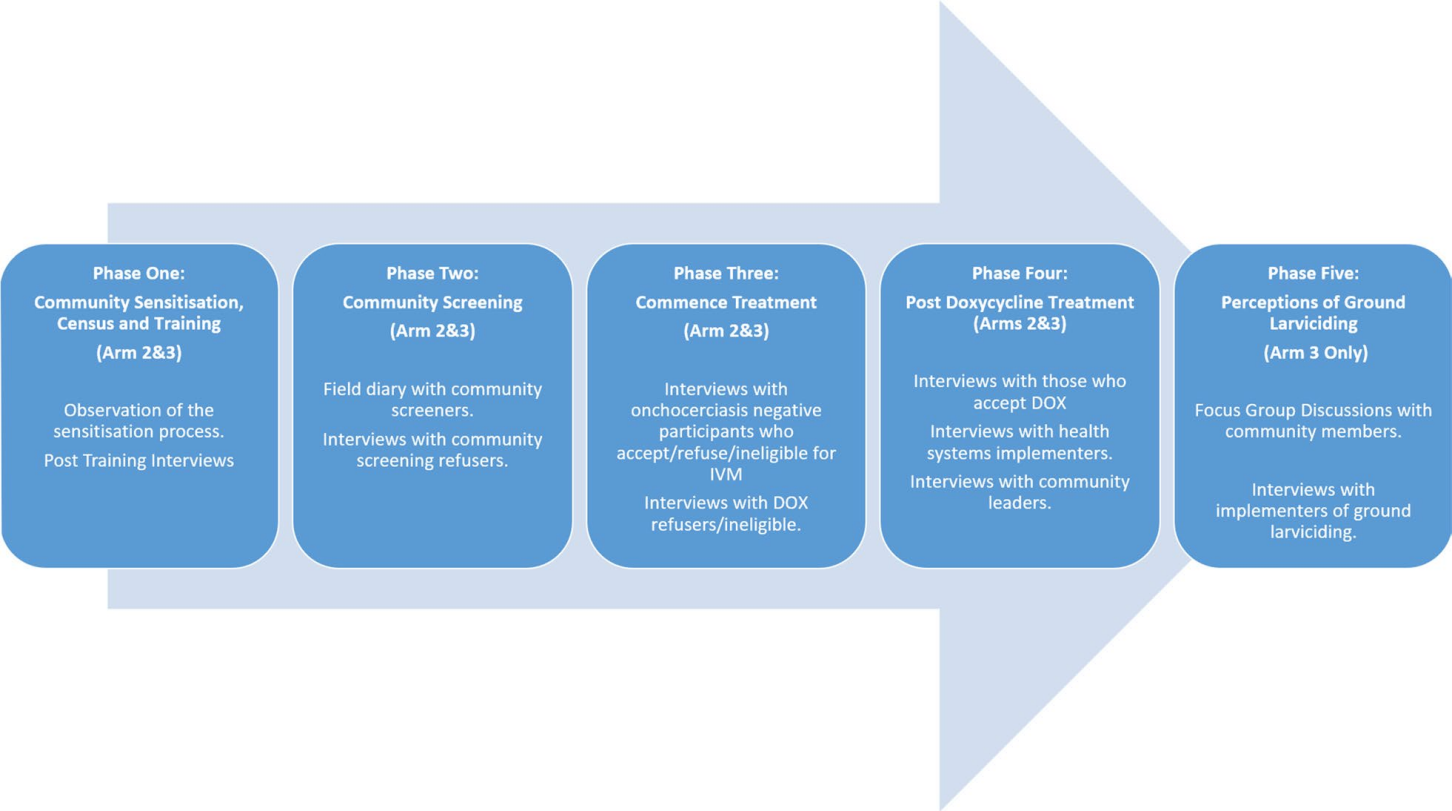
Overview of the qualitative study. Arm 1: control/enhanced monitoring/health systems as usual; Arm 2: test-and-treat with doxycycline; Arm 3: test-and-treat with doxycycline and vector control with ground larviciding. *Abbreviations*: DOX, doxycycline; IVM, ivermectin

An overview of the qualitative study phases is presented in Fig. 3.

#### Observation of implementation of the test-and-treat process

Using observation guides, members of the research team will observe and document the activities at the different workstations, capturing information pertaining to the behavioural patterns of the participants and other aspects of the research implementation.

#### Field diaries of community screeners

Community screeners (those who are involved in skin assessment, nodule palpation, skin snip or finger prick blood tests), will be asked to keep a field diary of their experience and observations when completing the community screening process, including details on challenges experienced, screening refusal, or support by community members. Four members of each community screening team (one per screening activity: skin assessment, nodule palpation, skin snip and finger prick blood test) will be asked to keep a field diary in the intervention study arms (Arms 2 and 3). Across all interview sub-categories, maximum variation in age and gender of respondent was aimed for from the total sample frame. At the end of the community screening phase, participants will be asked to talk through key elements of their observations.

### Semi-structured interviews

Between 8 and 16 interviews will be conducted with those who refuse at the community screening phase to understand current health related complaints that could be linked to onchocerciasis, experience of the CDTi programme, and reasons why they have refused to be part of the community screening for the alternative control strategy.

Between 8 and 16 interviews with people who are onchocerciasis-negative and refused CDTi will be conducted to explore reasons why people do or do not take IVM as well as their reaction to receiving a negative diagnosis for onchocerciasis.

Between 8 and 16 interviews with those who are onchocerciasis-positive but refuse DOX will be conducted to explore reasons why people refuse DOX.

Between 24 and 48 interviews with participants who accepted the DOX treatment will be conducted with those who agreed to take DOX to explore their experience of the DOX treatment and its impact on any pre-existing skin complaints as perceived by the participants.

Between 8 and 16 Key Informant interviews with influential community members will be completed to explore community perceptions of the intervention.

Between 8 and 16 semi-structured interviews will be conducted with CDDs and between 4 and 6 with Chief of Centres who were trained as part of the TTd control strategy to understand current community contexts in relation to onchocerciasis and CDTi, and to allow for the evaluation and strengthening of the training process.

Interviews with health systems implementers of the alternative control strategy will be conducted with CDDs (8–16 interviews) and Chief of Centres (4–6 interviews) following completion of the alternative control strategy to explore perceptions of the entire strategy from a health system implementers perspective.

Following completion of ground larviciding activities, between 4 and 8 interviews will be completed with individuals applying the treatment to the river. These interviews will explore how individuals were recruited, trained and what were their experiences during the intervention period.

#### Focus group discussions with community members about ground larviciding

Following completion of ground larviciding activities, focus group discussions will be completed with community members to understand perceptions of ground larviciding and the perception of insects and disease in the community. Groups will be conducted with men, women and youths.

#### Qualitative data analysis

Interviews and focus group discussions will be recorded and transcribed verbatim. The transcripts and field notes will be managed using AtlasTi (www.atlasti.com) or NVIVO 10 (QRS Software, Melbourne). A framework approach will be used to analyse the data which includes: (i) Familiarisation with the data during which time the team will read and re-read the data to identify common or recurring themes; (ii) Developing a thematic/coding framework-based on research aims and objectives and any inductive themes identified during the familiarisation process; and (iii) Indexing/coding data: the thematic/coding framework will be applied to all the data by the research team. This will then be followed by an explorative phase of charting the data [34]. The final stage will be mapping, which is a process to interpret and map the range of polarities and similarities within the data. All qualitative analyses will be completed by core members of the social science team, and analysis and findings discussed with a wider audience at key points within the study. This process will support in enhancing the trustworthiness of the research findings [34].

### Health economics specific methodology

Economic analyses of the proposed intervention will apply cost-effectiveness and cost-benefit analyses. The cost-effectiveness analysis will compare the intervention alternatives (TTd and TTd+VC) in their costs and effectiveness. Costs (investment) of intervention will be estimated from the societal perspective which includes costs of the intervention programme and costs borne by the beneficiaries of the programme.

Effectiveness will be measured in natural units such as the number of successfully treated patients, the number of life years gained, the number of symptom days averted, and number of deaths and disabilities averted. Incremental cost-effectiveness ratio will be calculated to estimate the cost for an additional outcome by employing TTd+VC in comparison with TTd alone. Further, cost-benefit analysis (CBA) will be conducted to assess the economic viability of TTD+VC. In CBA, benefits of the intervention programme in terms of total foregone cost-of-illness (monetary unit) due to reduced morbidity will be calculated and subtracted from the total cost of intervention for calculating the net benefits. If we get the value of the net benefit more than zero, the intervention will be considered as economically viable [35].

Structured questionnaires will be used for collecting data on the costs of intervention alternatives (TTd and TTd+VC). Budget and expenditure of the intervention programmes will be reviewed, and key management staff will be interviewed for identifying the cost items and their validation [36, 37]. For estimating the foregone costs of illness, i.e. benefits of the intervention in monetary unit) due to intervention, we shall collect data from relevant patients and their healthcare providers using structured pretested questionnaires. Direct medical (consultation, medicines, diagnostic tests) and non-medical costs (like, food, transport, lodging) as well as indirect costs (income loss of the patients and caregivers) will be captured in foregone Cost of Illness analysis (COI) [35].

## Discussion

Recent surveys indicate persistence of onchocerciasis transmission following 12 rounds at coverage ≥ 65% in South West Cameroon [7]. These data highlight that a transition from control to elimination with the current CDTi strategy is complicated by multiple factors. A low participation rate to annual IVM MDA has been identified in SW Cameroon, where self-reported adherence is significantly associated with onchocerciasis mf skin infection status and intensity of infection [7, 38–40]. Multiple societal factors culminate in inadequacy of adherence and/or participation ranging from IVM ‘treatment fatigue’, programmatic challenges faced the local health system and perceived risks of adverse reactions to IVM, including knowledge of *L. loa* related SAE experiences [8, 9, 41].

Two WHO-endorsed alternative approaches, with the potential to circumvent IVM MDA-specific drawbacks, are: oral treatment with the macrofilaricide, DOX and localised ground-larviciding vector control with temephos. Whilst prior implementations of both have demonstrated their potential to accelerate onchocerciasis elimination, the parasitological and transmission impact of combining the two strategies has not before been evaluated [18, 20, 42]. Further, societal acceptability of these approaches in an area of low CDTi participation has not been detailed. Further, the financial feasibility of implementing either of these approaches has not been prior interrogated.

Here we have detailed the Department for International Development (DfID) funded COUNTDOWN inter-disciplinary research protocol for implementation of a test-and-treat with DOX alone or combined with temephos ground larviciding.

We hypothesise that through offering alternative approaches to IVM and engaging communities throughout the process there will be lasting positive impacts that will contribute to addressing some of the challenges and concerns faced by communities. We hypothesise that by offering these alternatives to IVM, the poor community perception toward onchocerciasis elimination can be overcome. Further, we predict that a sufficient uptake of DOX treatment, a macrofilaricide with a superior range of anti-filarial efficacies which does not cause loiasis-associated SAE, potentially in combination with vector control, will result in a significant (minimum 37%) decline in community prevalence of *O. volvulus* skin infection. The cost of implementation will be accurately recorded and reported, along with patient and health care worker perceptions of the interventions, so that future elimination programmes can consider the acceptability and feasibility of scaling up these approaches.

## Data Availability

Not applicable. The data will be made freely and publicly available at the completion of the studies and analysis of study outcomes.

## Abbreviations

AE: adverse event
APOC: African Programme for Onchocerciasis Control
CDTi: community-directed treatment with ivermectin
COI: cost of illness analysis
DOX: doxycycline
ESPEN: Extended Special Programme for the Elimination of NTDs
GPS: global positioning system
IVM: ivermectin
LSTM: Liverpool School of Tropical Medicine
MDA: mass drug administration
mf: microfilariae
NTD: neglected tropical disease
OCP: onchocerciasis control programme
TTd: test-and-treat with doxycycline
SAE: serious adverse event
WHO: World Health Organization
